# Rhenium Electrodeposition and Its Electrochemical Behavior in Molten KF-KBF_4_-B_2_O_3_-KReO_4_

**DOI:** 10.3390/ma15238679

**Published:** 2022-12-05

**Authors:** Aleksandr A. Chernyshev, Stepan P. Arkhipov, Alexey P. Apisarov, Aleksander S. Shmygalev, Andrey V. Isakov, Yury P. Zaikov

**Affiliations:** Institute of High Temperature Electrochemistry, Ural Branch, Russian Academy of Sciences, 20 Akademicheskaya Street, 620066 Ekatherinburg, Russia

**Keywords:** cathodic behavior, rhenium, electrochemical analysis, molten salts

## Abstract

The electrochemical behavior of rhenium ions in the molten KF-KBF_4_-B_2_O_3_ salt was systematically studied, and pure metallic rhenium was obtained at the cathode. The processes of rhenium ions reduction and diffusion in molten KF-KBF_4_-B_2_O_3_ were determined using cyclic voltammetry, stationary galvanostatic and polarization curves analyses. The values of diffusion coefficients were 3.15 × 10^−5^ cm^2^/s and 4.61 × 10^−5^ cm^2^/s for R_1_ and R_2,_ respectively. Rhenium electrodeposition was carried out at a constant potential. The process of rhenium cathode reduction in KF-KBF_4_-B_2_O_3_ at 773 K was found to be a one-step reaction Re(VII) → Re, and rhenium electrodeposition presumably occurred from two types of complex rhenium ions (KReO_4_ and K_3_ReO_5_). Both processes are quasi-reversible and controlled by diffusion. The obtained cathode deposit was analyzed by SEM, EDX, ICP-OES and XRD methods. The obtained deposit had a thread structure and rhenium was the main component.

## 1. Introduction

Rhenium was discovered in 1925 and it is considered to be an extremely rare metal with unique properties. Despite low rhenium concentration in the earth’s crust of about 10^−7^%, it acquired wide industrial application [1,2]. World rhenium consumption from primary sources ranges from about 50 to 60 metric tons per year [3]. About 70% of the excavated rhenium is used for the production of superalloys for the aerospace industry. About 20% of rhenium is used as bimetallic Pt-Re catalysts in the oil-processing industry for the production of high-octane hydrocarbons used for lead-free gasoline production. The remaining 10% of rhenium is used as Re-W and Re-Mo alloys for the production of electrical contact points, light globes, heating elements, vacuum pipes and X-ray tubes [1,4].

To date, only a few methods of metallic rhenium production are known. The method of rhenium oxides and/or potassium and ammonium perrhenates reduction by hydrogen is the most common [5,6]. Rhenium-based coatings may be obtained by the electrolysis of aqueous solutions of perrhenates and chlorrhenates [7,8,9,10,11,12,13]. Despite their drawbacks, the described methods are widely used. For instance, it is impossible to obtain compact rhenium deposits by the hydrogen reduction method. It is possible to obtain coatings composed of compact rhenium by the aqueous solutions electrolysis, but the thickness of the obtained deposits is about 10 µm.

The method of molten salts electrolysis is the most beneficial method of metallic rhenium and rhenium-based alloys production. This method has a number of advantages, as opposed to other known methods of rhenium production; it allows obtaining rhenium in metallic and powder form; compact, plastic, non-porous and coherent rhenium layers may be formed on a complex large-scale substrate. Chloride [14,15,16,17], fluoride [18,19], chloride–fluoride [20] and fluoride–borate melts [2,21,22] may be used as electrolytes.

We have previously studied the mechanism of rhenium electroreduction on the rhenium substrate in the fluoride–borate melt containing potassium perrhenate. Compact rhenium alloys of 99.98 wt.% purity were obtained. The initial stages of rhenium electrocrystallization form the KF-KBF_4_-B_2_O_3_-KReO_4_ melt on a glassy carbon substrate were studied.

The present paper is devoted to the study of the rhenium cathode reduction processes on the glassy-carbon and platinum substrates. The present research elucidates the possibility of forming metallic rhenium nanofibers in the KF-KBF_4_-B_2_O_3_-KReO_4_ melt.

## 2. Materials and Methods

The KF(37.28 wt.%)-KBF_4_(40.39 wt.%)-B_2_O_3_(22.33 wt.%) melt was prepared in a glassy-carbon container by fusing individual salts. Commercial chemically pure individual KF, KBF_4_ and B_2_O_3_ salts were provided by Vekton Company, Saints-Petersburg, Russia. The obtained melt was heated to 772 K and exposed in molten state for 4 h to remove the fluoric acid. The residual moisture was removed by the galvanostatic electrolysis at the current load of 0.003 A/cm^2^. The electrolysis was performed until hydrogen evolution at the cathode stopped. The visual examination of the melt optical transparency was used to determine the quality of HF and moisture removal. The rhenium concentration (1–6 wt.%) in the melt was set by direct addition of potassium perinate powder. The chemical composition of the prepared melts was controlled by the method of optic emission spectrometry with the inductively coupled plasma using an iCAP 6300 Duo device (Thermo Scientific, Waltham, MA, USA).

**Figure 1:**
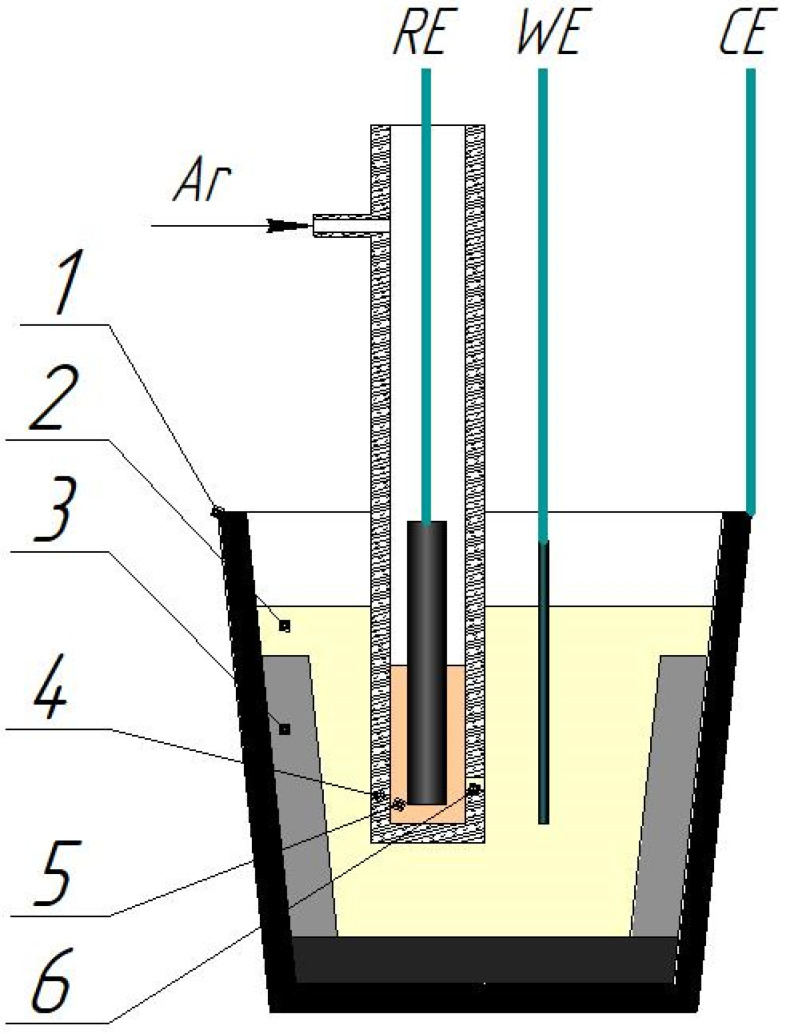
Schematic of the electrochemical cell: 1—glassy carbon reactor crucible; 2—studied electrolyte; 3—rhenium rod (counter electrode); 4—BN body of the reference electrode; 5—KF-KBF4-B2O3-KReO4 (6 wt.%) melt; 6—capillary composed of porous BN.

Voltammetry measurements were performed in a three-electrode electrochemical cell (Figure 1) in the ambient air atmosphere. The cell was assembled in a glassy carbon container (GC-2000) of 600 cm^3^ in volume. Rhenium rods of 99.9 wt.% purity and ~85 cm^2^ square were used as auxiliary electrodes. Glassy carbon and platinum rods of 1.9 mm and 1 mm in diameter and 0.8 cm^2^ and 0.36 cm^2^ in square, respectively, served as working electrodes. A rhenium electrode served as a reference electrode; it was immersed into the KF-KBF_4_-B_2_O_3_-KReO_4_ (6 wt.%) melt in the argon atmosphere (99.998 wt.%). The electrolyte of the reference electrode and the studied melt were separated by a diaphragm composed of pyrolytic boron nitride. The measurements were performed using a galvanostate-potentiostate Autolab PGStat 302N (Metrohm, Herisau, Switzerland) with the Nova 2.1.4 software (Metrohm Autolab B.V., Utrecht, The Netherlands). The ohmic losses were controlled and compensated via Autolab.

To determine the chemical composition, the obtained cathode deposits were analyzed using the methods of optic emission spectrometry with the inductively coupled plasma iCAP 6300 Duo (Thermo Scientific, USA); X-ray diffraction method using a Rigaku D/MAX 2200VL/PC diffractometer (Rigaku, Tokyo, Japan); scanning electron microscopy using Tescan Vega 4 with the EDX Oxford Xplore 30 system (Tescan, Brno, Czech Republic). The oxygen concentration in the cathode deposit was determined by the method of reductive melting in the bearer gas flow using the nitrogen and oxygen analyzer Metavack-K (SPO”EKSAN”, Izhevsk, Udmurt Republic, Russia).

## 3. Results and Discussion

### 3.1. Electrochemical Behavior of Rhenium Ions in Molten KF-KBF_4_-B_2_O_3_

**Figure 2:**
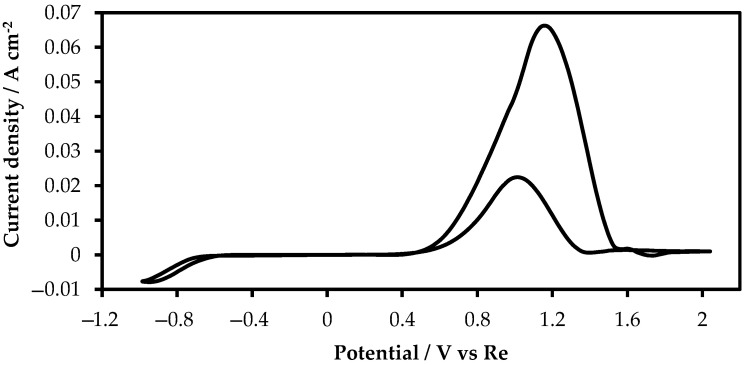
Electrochemical window of molten KF-KBF_4_-B_2_O_3_ on a glassy carbon wire electrode; Scan rate: 250 mV/s; RE:Re; CE:GC.

First, we performed cyclic voltammetry measurements using a glassy carbon electrode with a scanning rate of 250 mV/s at 773 K in molten KF-KBF_4_-B_2_O_3_ without rhenium ions. This experiment was carried out to identify the electrochemical window. The obtained cycling voltammetry is illustrated in Figure 2.

Extremely low current densities were recorded within the interval of potentials of 0.50 to −0.70 V, relative to the rhenium reference electrode. No clear current density peaks were detected, which elucidates that there were not any redox reactions in the analyzed region of potentials. In the region of potentials more positive than 0.5 V, a clear peak is observed, which is characterized by the reaction of carbon dioxide evolution.

**Figure 3:**
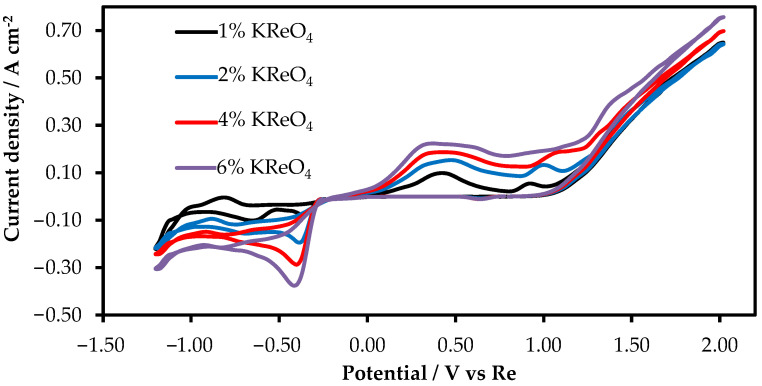
Cyclic voltammogram of KReO_4_ in molten KF-KBF_4_-B_2_O_3_ on a platinum wire electrode. Scan rate: 100 mV/s; RE:Re; CE:Re.

To obtain information on the behavior of rhenium ions, we recorded cyclic voltammetry patterns with a scan rate of 100 mV/s on the glassy carbon and platinum wire electrode after the KReO_4_ addition to the KF-KBF_4_-B_2_O_3_ melt. The voltammetry pattern is illustrated in Figure 3. At the KReO_4_ concentration of 1 wt.%, two redox peaks appeared. This may illustrate that the rhenium ions electroreduction is a two-stage process. However, as the KReO_4_ concentration increased up to 6 wt.%, the second peak of rhenium reduction was not recorded at the rate of the potential change of 100 mV/s.

**Figure 4:**
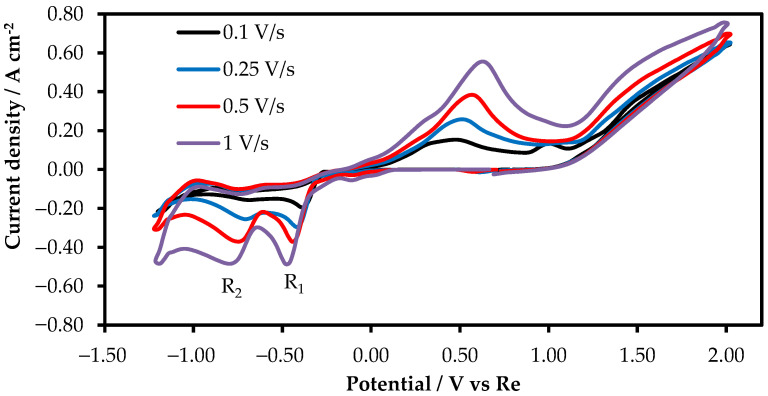
Cyclic voltammogram of 2 wt.% of KReO_4_ in molten KF-KBF_4_-B_2_O_3_ on a platinum wire electrode. RE:Re; CE:Re.

To study the electrochemical behavior of rhenium ions in the KF-KBF_4_-B_2_O_3_ melt, the cyclic voltammetry was performed at different scan rates. The recorded voltammograms are presented in Figure 4.

**Figure 5:**
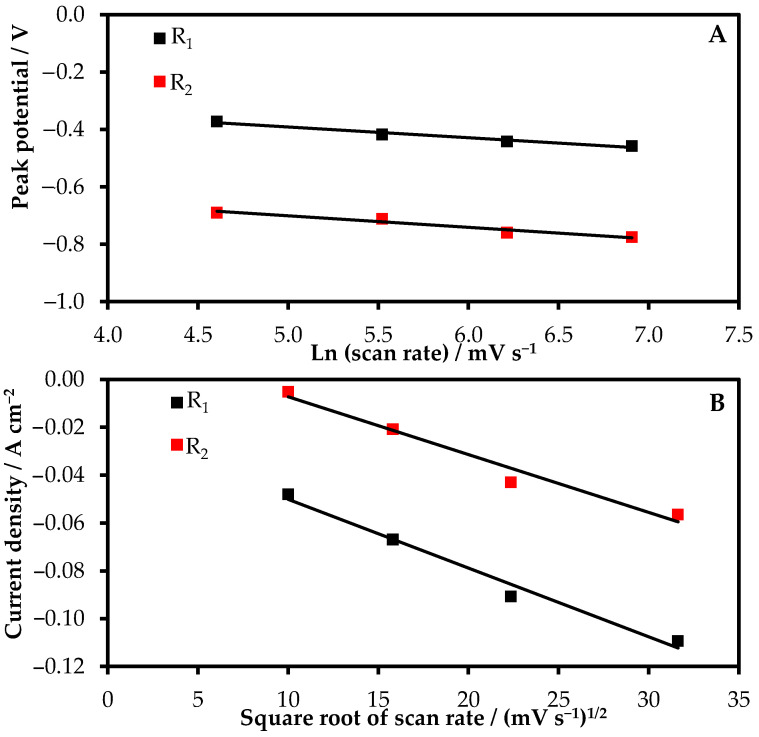
The dependences of the cathodic peak potential on the logarithm of the scan rate (**A**); the relationship between the current density and the square root of scan rate (**B**).

As the rate of the potential change increased, the second peak of rhenium reduction became clearer. The reduction potentials R_1_ and R_2_ changed insignificantly as the scan rate increased (Figure 5A), which testified that R_1_ and R_2_ denote quasi-reversible processes. To determine the limiting stage of R_1_ and R_2_ reduction, the relation between the peak values of the current densities of R_1_ and R_2_ and square roots of the potential change rates were studied. Figure 5B demonstrates that R_1_ and R_2_ are linearly dependent, which suggests that rhenium reduction is a diffusion-controlled reaction.

**Figure 6:**
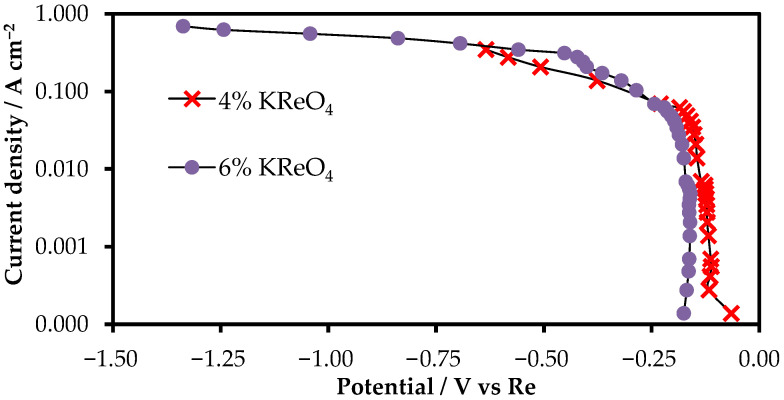
Stationary polarization curves of KReO_4_ in molten KF-KBF_4_-B_2_O_3_ on a platinum wire electrode. RE:Re; CE:Re.

The method of stationary galvanostatic polarization curves demonstrated that the process of rhenium reduction was accompanied with the transfer of seven electrons. The stationary polarization curves are presented in Figure 6. A negligible deviation of the potential from the equilibrium value was observed on the initial region of the polarization curve at the increase in the cathode current density up to 0.05 A/cm^2^. The experimental dependence of potential on the current density logarithm falls on straight lines in this region. The values of the tangents of the inclination angle of the dependence of the potential on the current density logarithm were 0.0092 and 0.0095 for 4 and 6 wt.% of KReO_4_, respectively. The number of electrons participating in reaction of the cathode reduction was calculated using Equation (1):

(1) $$n=\frac{RT}{tg\alpha \cdot F}$$

the calculated values of *n* were 7.23 and 7.01 for 4 and 6 wt.% of KReO_4_, respectively. Therefore, the process of rhenium cathode reduction is accompanied with the transfer of seven electrons.

**Figure 7:**
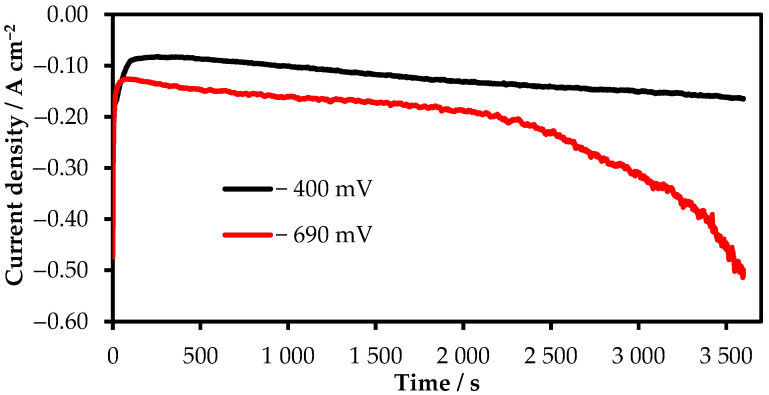
Chronoamperogram of potentiostatic electrolysis of 6 wt.% KReO_4_ in molten KF-KBF_4_-B_2_O_3_ on a platinum wire electrode. RE:Re; CE:Re.

As the R_1_ and R_2_ processes are quasi-reversible, to verify the amount of electrons participating in the reduction reaction determined by the method of stationary polarization curves, we performed additional research by the potentiostatic electrolysis. To determine the number of electrons for R_1_ and R_2_, the electrolysis was performed at the cathode overpotential of 400 mV and 690 mV, respectively. In the course of electrolysis, rhenium deposits were obtained in the amount of 0.2283 g and 0.7629 g at 400 and 690 mV, respectively. According to the obtained chronopotentiogramms, presented in Figure 7, the amount of the passed electricity was calculated. The number of electrons in the process was calculated according to the amount of the electricity passed using Faraday’s law. It was determined that the reduction reactions during both R_1_ and R_2_ processes are accompanied with seven electrons.

Due to the fact the maximum rhenium oxidation degree is VII, the presence of two reduction peaks, accompanied with the transfer of seven electrons, testifies that two types of Re(VII) ions discharge. We may assume that during the cathode reduction of KReO_4_, the conditions for K_3_ReO_5_ synthesis appear. At the cathode polarization up to 400 mV, rhenium deposits from KReO_4_, and at the cathode polarization exceeding 400 mV, the process becomes limited by diffusion. The near-electrode layer is depleted of rhenium ions and K_3_ReO_5_ forms; the cathode process may be described by Equation (2):

(2) 3 ReO4− + 7 e− → Re + 2 ReO53− + 2 O2−

At the further electrode polarization, rhenium reduces from both KReO_4_ (Equation (2)) and K_3_ReO_5_ (Equation (3)).

(3) ReO53− + 7 e− → Re + 5 O2−

The diffusion coefficient of Re(VII) ions in molten KF-KBF_4_-B_2_O_3_ can be calculated according to Equation (4) [23,24]:

(4) ip=0.4463⋅n⋅F⋅C⋅(nFRT)1/2D1/2υ1/2

where i_p_ is the peak current density (mA/sm^2^), ν is the scan rate (mV/s), C is the bulk concentration of the reducible ion (mol/cm^3^) and D is the diffusion coefficient (cm^2^/s).

The calculated diffusion coefficient of Re(VII) ions in molten KF-KBF_4_-B_2_O_3_ with 2wt.% KReO_4_ at 773 K was 3.15 × 10^−5^ cm^2^/s and 4.61 × 10^−5^ cm^2^/s for R_1_ and R_2_, respectively.

### 3.2. Potentiostatic Electrolysis

**Figure 8:**
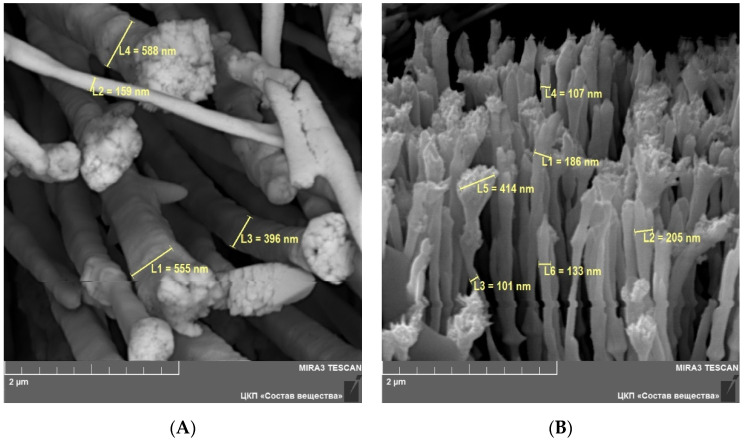
SEM images of rhenium nanofibers obtained at the potentials of 400 mV (**A**) and at the potentials of 690 mV (**B**).

During the potentiostatic electrolysis rhenium was formed at the surface of the glassy carbon crucible in the form of nanofibers of 100–600 nm in diameter (Figure 8). The length of the nanofibers was about 150–200 µm. The reduction was performed at the potentials of 400 mV and 690 mV. During the electrolysis, we obtained metallic rhenium nanofibers of 200–600 nm (Figure 8A) and 100–200 nm (Figure 8B) in diameter, respectively.

**Figure 9:**
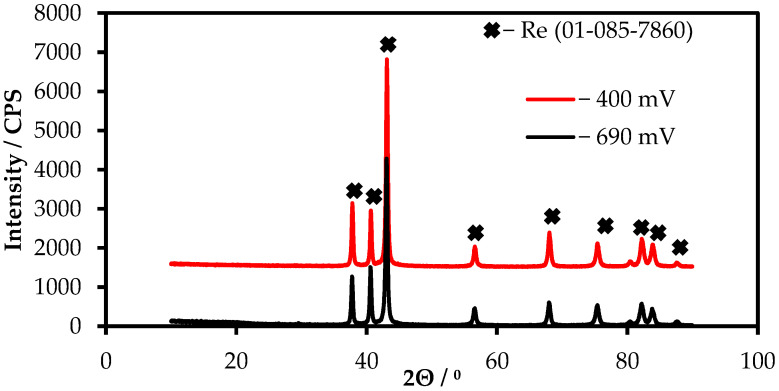
XRD diffraction pattern of the rhenium deposit obtained during the electrolysis in the KF-KBF_4_-B_2_O_3_-KReO_4_ melt.

The X-ray diffraction analysis of the obtained nanofibers demonstrated that the deposit has a homogeneous rhenium phase (Figure 9). Other types of phases were not determined.

**Figure 10:**
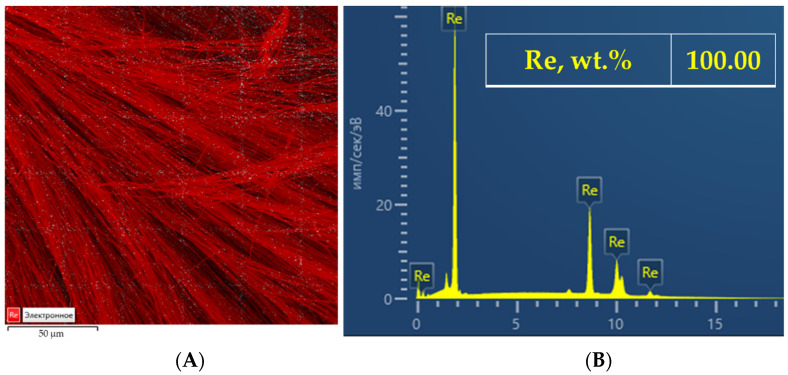
Distribution map Re (**A**); the result of EDX analysis (**B**).

The energy-dispersive X-ray spectroscopy analysis elucidated that rhenium was homogeneously distributed in the samples. The presence of any another metallic inclusions was not detected (Figure 10). The method of reduction melting demonstrated that the obtained rhenium deposits contained 0.5243 ± 0.0026 wt.% of oxygen. The presence of oxygen is probably associated with the removal of the molten salt residues from the surface of the deposit by rinsing in the deionized water. The obtained deposit was analyzed for the concentration of admixtures using the method of optic emission spectrometry with the inductively coupled plasma iCAP 6300 Duo (Table 1).

During the electrolysis, the electricity amounting to 830 C and 2772 C was passed via the electrochemical cell at the potential of 400 mV and 690 mV, respectively. According to the results of the gravimetric analysis, we calculated the actual current efficiency, which value was 100% for the current loads of both 400 mV and 690 mV.

The analysis of the chemical composition of the obtained deposits illustrated that it is possible to obtain metallic rhenium of the 99.98 wt.% purity by the electrolysis of the KF-KBF_4_-B_2_O_3_-KReO_4_ melts in air. Potassium is the main admixture because it is an integral parts of the melt cation sublattice. It should be mentioned that the boron concentration in rhenium is less than the method determination limit. This may be explained by the high temperatures of rhenium and boron interaction.

## 4. Conclusions

The electrochemical behavior of Re(VII) in molten KF-KBF_4_-B_2_O_3_ was systematically studied in a three-electrode electrochemical cell at 773 K. It was found that the process of rhenium ions reduction is a quasi-reversible reaction controlled by diffusion. Rhenium was found to reduce from two types of seven valency ions. First, rhenium preferably reduced from the potassium perrhenate, and, at the further polarization, the reduction proceeded from the mixture of potassium perrhenate and mesa perrhenate. Diffusion coefficients of Re(VII) corresponded to its reduction from KReO_4_ and KReO_4_ + K_3_ReO_5_, were analyzed using the method of cyclic voltammetry. Their values were 3.15 × 10^−5^ cm^2^/s and 4.61 × 10^−5^ cm^2^/s, respectively.

The nano-fibers of metallic rhenium on the glassy carbon substrate were obtained during the potentiostatic electrolysis of the KF-KBF_4_-B_2_O_3_ melt. The formed deposits were analyzed by the SEM with EDX and ICP-OES methods. The obtained results testified that the obtained deposits were composed of highly pure metallic rhenium (99.98%).

## Figures and Tables

**Table 1:** Content of elements in the obtained deposits.

Content of Elements, wt.%										
Fe	Mg	Si	Ni	Mo	Cu	Al	Ca	K	B	Re
0.0010	0.0002	0.0011	0.0010	0.0010	0.0005	0.0007	0.0010	0.0110	0.0077	99.9788
